# Data for automated, high-throughput microscopy analysis of intracellular bacterial colonies using spot detection

**DOI:** 10.1016/j.dib.2017.08.027

**Published:** 2017-09-01

**Authors:** Christina L. Ernstsen, Frédéric H. Login, Helene H. Jensen, Rikke Nørregaard, Jakob Møller-Jensen, Lene N. Nejsum

**Affiliations:** a Department of Clinical Medicine, Aarhus University, 8000 Aarhus, Denmark; b Department of Molecular Biology and Genetics, Aarhus University, 8000 Aarhus, Denmark; c Department of Biochemistry and Molecular Biology, University of Southern Denmark, 5230 Odense M, Denmark

**Keywords:** Uropathogenic *E. coli*, UPEC, Invasive bacteria, Intracellular bacterial colonies, High-throughput imaging, Spot detection

## Abstract

Quantification of intracellular bacterial colonies is useful in strategies directed against bacterial attachment, subsequent cellular invasion and intracellular proliferation. An automated, high-throughput microscopy-method was established to quantify the number and size of intracellular bacterial colonies in infected host cells (Detection and quantification of intracellular bacterial colonies by automated, high-throughput microscopy, Ernstsen et al., 2017 [1]). The infected cells were imaged with a 10× objective and number of intracellular bacterial colonies, their size distribution and the number of cell nuclei were automatically quantified using a spot detection-tool. The spot detection-output was exported to Excel, where data analysis was performed. In this article, micrographs and spot detection data are made available to facilitate implementation of the method.

**Specifications Table**

**Table:** 

Subject area	*Cell biology, microbiology*
More specific subject area	*Quantification of invasive colony forming pathogens*
Type of data	*Fluorescent images, Excel sheet with spot detection data*
How data was acquired	*High-throughput widefield microscopy (Nikon Ti Eclipse inverted fluorescence microscope). Spot detection (NIS Elements microscope imaging software (Nikon, version 4.51))*
Data format	*Raw image micrographs, spot detection data in Excel format*
Experimental factors	*UPEC CFT073 were transformed with TurboGFP (Evrogen)*
Experimental features	*Quantification of invasive colony forming pathogens*
Data source location	*NA*
Data accessibility	*All data are available*
Related research article	*Detection and quantification of intracellular bacterial colonies by automated, high-throughput microscopy (Journal of Microbiological Methods, 2017**[1]**)*

**Value of the data**

• The dataset provides raw data used to quantify and analyze intracellular bacterial colonies within host cells.
• Automated, high-throughput microscopy followed by spot detection determines the number of intracellular bacterial colonies, their size distribution and the average number per host cells.
• The quantification of intracellular bacterial colonies is useful in strategies directed against bacterial attachment, cellular invasion and intracellular proliferation.

## 1 Data

The data in this article provides information and raw data for detection and quantification of intracellular bacterial colonies by automated, high-throughput microscopy [1].

**Fig. 1:**
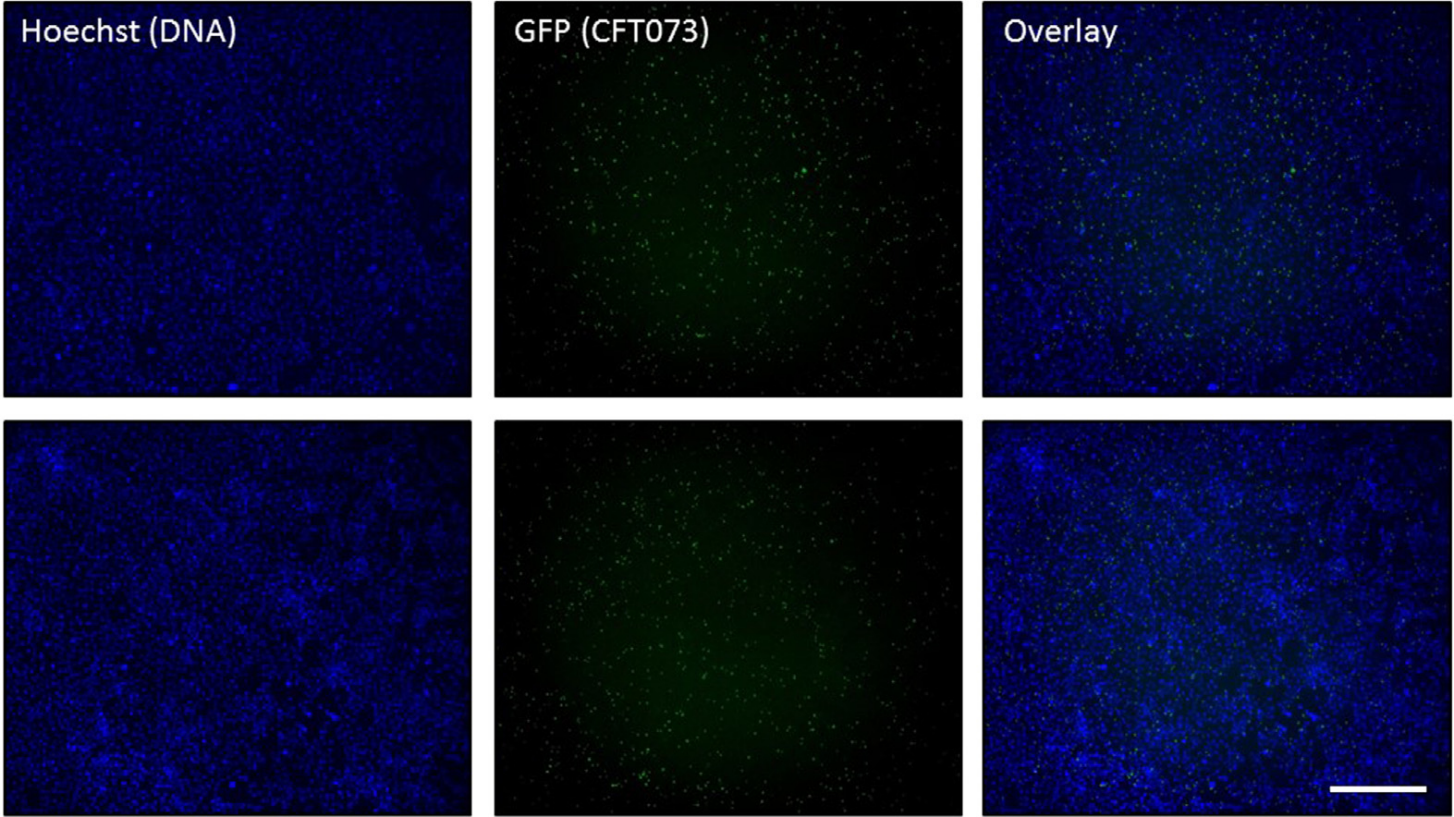
Full-size images of intracellular bacterial colonies of GFP-expressing *E. coli* CFT073 during infection of HKC-8 cells. Imaging was performed with a 10× objective using filters for Hoechst to visualize cell nuclei (blue) and GFP to visualize intracellular bacterial colonies (green). Scale bar is 400 µm.

**Fig. 2:**
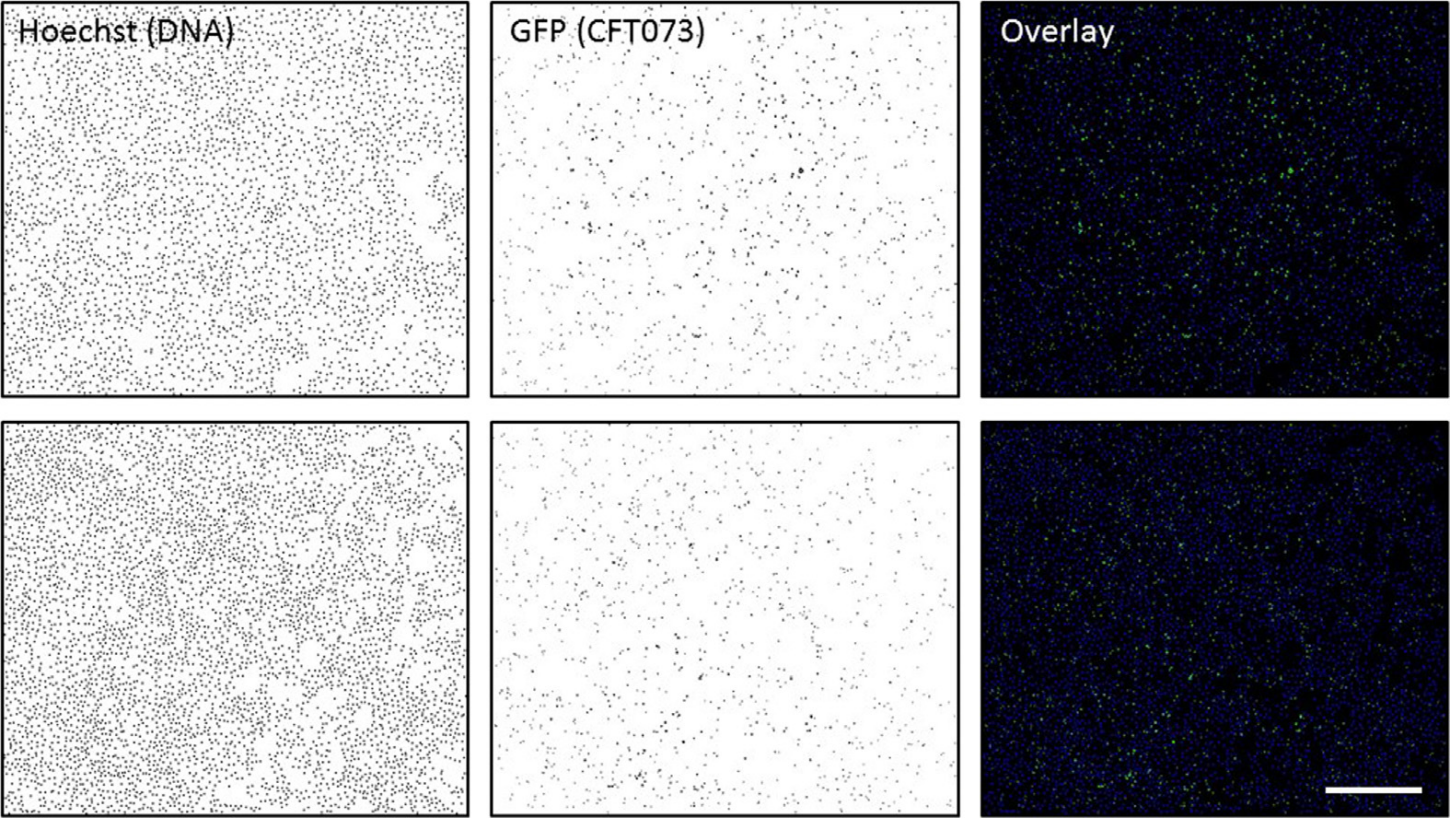
Binary images showing all identified cell nuclei and intracellular bacterial colonies, which were obtained by Automated Spot Detection of the images shown in Fig. 1. The binary images of Hochst and GFP are shown in grey scale, while overlay of the binary images are shown in color to distinguish between cell nuclei and intracellular bacterial colonies. Scale bar is 400 µm.

Fig. 1 shows full-size images of intracellular bacterial colonies formed by GFP-expressing *Escherichia coli* CFT073 during infection of human kidney cells (HKC-8) from two different wells from an experiment performed in a 48 well plate. Imaging was performed with a 10× objective using filters for Hoechst and GFP to visualize cell nuclei and intracellular bacterial colonies, respectively. Cell nuclei were visualized as blue spots and the intracellular bacterial colonies as green spots. Original micrographs (tif-format) of GFP-expressing intracellular bacterial colonies and Hoechst-stained cell nuclei are available for download (Supplementary micrographs 1–4). Supplementary original micrographs must be opened using an appropriate program for image handling like ImageJ [2]. Fig. 2 shows binary images containing all identified cell nuclei and intracellular bacterial colonies, which were obtained by Automated Spot Detection (NIS-Elements) of the images shown in Fig. 1. The number and size of intracellular bacterial colonies and the number of cell nuclei per image were quantified using the “Automated Measurement Results”-function. The spot detection-output were directly exported to Excel, where the data analysis was performed. The spot detection-output (number of objects and area) exported to Excel is available for download (Supplementary Excel data sheet).

## 2 Experimental design, materials and methods

### 2.1 Infection model

Human proximal tubular epithelial cells (HKC-8) [3] were seeded in 48 well plates and infected with GFP expressing uropathogenic *E. coli* (UPEC) CFT073 [4] at a multiplicity of infection (MOI) of 50. The plates were centrifuged for 5 min at 150×*g* and incubated at 37 °C with 5% CO_2_ for 1 h. To eliminate extracellular bacteria, infected cells were washed twice and incubated with 50 µg/mL gentamicin at 37 °C with 5% CO_2_ for 2 h before fixation and microscopic analysis.

### 2.2 Fluorescent staining and imaging

Cells and bacteria were washed with PBS, fixed with 4% paraformaldehyde, permeabilized with 0.1% Triton X-100 and 3% BSA in PBS and stained with Hoechst.

Images were acquired on a Nikon T*i* Eclipse inverted fluorescence microscope equipped with a pE-300 WHITE LED illumination unit, a Perfect Focus 3 system and 10× (NA 0.30) objective. Acquisition was performed with an Andor Zyla 5.5 Mpixel camera (Andor Technology Ltd., Belfast, UK). For visualization of Hoechst a 340–380 excitation filter and a 435–485 emission filter were used, and for visualization of GFP a 469/35 excitation filter and a 525/39 emission filter were used.

### 2.3 Image analysis and spot detection

NIS Elements microscope imaging software (Nikon, version 4.51) was used to quantify the number and size of intracellular bacterial communities within infected cells. GFP-expressing bacteria, visible as bright green spots in images acquired with a 10× objective, were detected, counted and measured using the software Automated Spot Detection. The settings of Automated Spot Detection were set in order to detect all bright spots with a”Typical diameter” of 6 μm and with a”Contrast” of 80. The function”Detect all objects” was activated. The intracellular bacterial colonies were identified and selected with a selection of 1 pixel binary, and by using the”Growing”-function (set to 2800), the size of each binary region was expanded to the edges of all detected intracellular bacterial colonies. The Automated Spot Detection gave a binary image identifying all GFP expressing bacteria in each image frame within the defined settings. By using the “Automated Measurement Results”-function, the number and size distribution of intracellular bacterial colonies in each image were quantified and afterwards exported to Excel. To calculate the number of intracellular bacterial colonies per host cell, the number of HKC-8 was determined using a similar approach. HKC-8 cell nuclei were visualized by Hoechst staining, and identified as bright spots with a “Typical diameter” of 12 μm and with a “Contrast” of 10. Again, the number of cells per image was quantified using the “Automated Measurement Results”-function, which afterwards was exported to Excel.

Analyses of the measured numbers and sizes were performed in Excel, and preparation of images for visual inspection and presentation was performed using ImageJ software [2].

## Funding

This work was supported by a Lundbeck Junior Group Leader Fellowship : R37-A3461 to LNN from the Lundbeck Foundation and Aarhus University Research Foundation: AUFF-E-2015-FLS-8-5-HD to LNN. The Nikon microscope was funded by the Carlsberg Foundation: 2013_01_0566.

## Figures and Tables

**Fig. S1:**
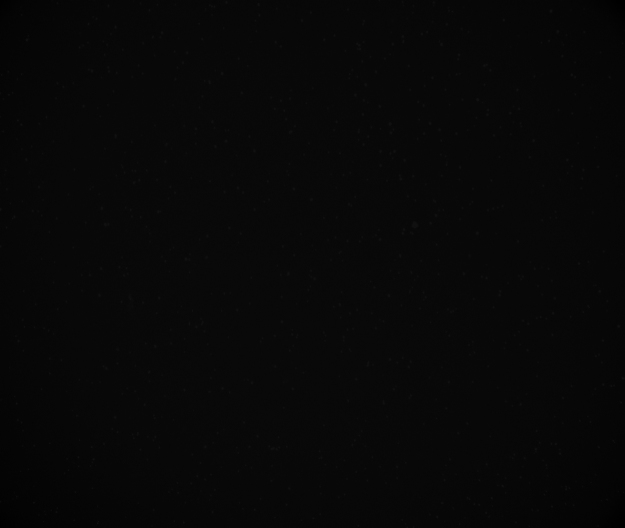
*Supplementary micrograph 1. Micrograph of GFP-expressing E. Coli CFT073 from experiment 1, well 1. Note that the image must be opened in an appropriate image analysis program like ImageJ**[2]*.

**Fig. S2:**
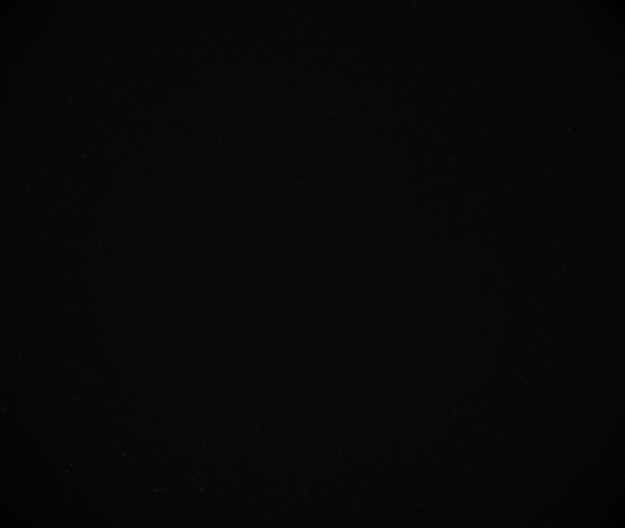
*Supplementary micrograph 2. Micrograph of GFP-expressing E. Coli CFT073 from experiment 1, well 2. Note that the image must be opened in an appropriate image analysis program like ImageJ**[2]*.

**Fig. S3:**
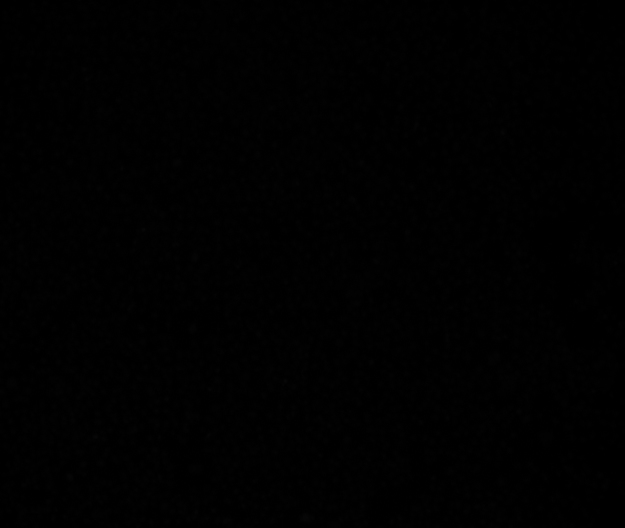
*Supplementary micrograph 3. Micrograph of Hoechst (cell nuclei) from experiment 1, well 1. Note that the image must be opened in an appropriate image analysis program like ImageJ**[2]*.

**Fig. S4:**
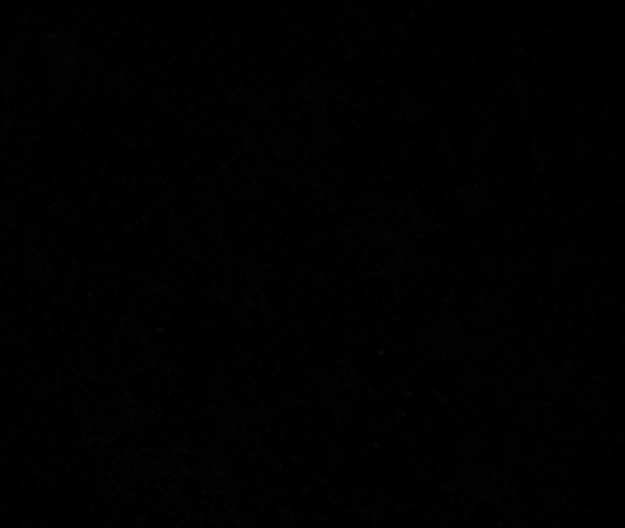
*Supplementary micrograph 4. Micrograph of Hoechst (cell nuclei) from experiment 1, well 2. Note that the image must be opened in an appropriate image analysis program like ImageJ**[2]*.

## Appendix A Supplementary material

.

Supplementary Excel data sheet. The number and size of intracellular bacterial colonies and the number of cell nuclei per image were quantified using the “Automated Measurement Results”-function. Data were exported to Excel. Supplementary material
